# Prolonged grief disorder in an inpatient psychiatric sample: psychometric properties of a new clinical interview and preliminary prevalence

**DOI:** 10.1186/s12888-024-05784-2

**Published:** 2024-05-01

**Authors:** Mirjam Sophie Rueger, Franziska Lechner-Meichsner, Lotte Kirschbaum, Silke Lubik, Sibylle C. Roll, Regina Steil

**Affiliations:** 1 Department Clinical Psychology & Psychotherapy, Goethe-University Frankfurt am Main, Varrentrappstraße 40-42, 60486 Frankfurt am Main, Germany; 2 Department of Clinical Psychology, Utrecht University, Heidelberglaan 8, Utrecht, 3584 CS The Netherlands; 3 Klinik für psychische Gesundheit, varisano Klinikum Frankfurt Höchst, Gotenstraße 6-8, 65929 Frankfurt am Main, Germany

**Keywords:** Prolonged grief disorder, Inpatient sample, Prevalence, Clinical interview

## Abstract

**Background:**

Prolonged Grief Disorder (PGD) was newly included in the ICD-11 and DSM-5-TR. It is not yet part of the standard assessments in many healthcare systems, including psychiatric wards. Because disordered grief is associated with suicidality, sleep problems and substance use disorders, an investigation into PGD in psychiatric inpatients is warranted.

**Method:**

We interviewed *N* = 101 psychiatric inpatients who were admitted to the open psychiatric wards and the day hospital of a German psychiatric hospital and who had lost a person close to them. Assessments comprised clinical interviews and self-report instruments covering PGD and other mental disorders. We specifically developed the International Interview for Prolonged Grief Disorder according to ICD-11 (I-PGD-11) for the study and examined its psychometric properties.

**Results:**

The prevalence rate of PGD among bereaved patients according to ICD-11 was 16.83% and according to DSM-5-TR 10.89%. The I-PGD-11 showed good psychometric properties (Mc Donald’s ω = 0.89, ICC = 0.985). Being female, having lost a child or spouse, and unnatural or surprising circumstances of the death were associated with higher PGD scores.

**Trial registration:**

Approval was obtained by the ethics committee of the of the Goethe University Frankfurt (2021-62, 2023-17) and the Chamber of Hessian Physicians (2021-2730-evBO). The study was preregistered (10.17605/OSF.IO/K98MF).

**Limitations:**

We only assessed inpatients of one psychiatric clinic in Germany, limiting the generalizability of our findings.

**Conclusion:**

The present study underlines the importance of exploring loss and grief in psychiatric inpatients and including PGD in the assessments. Given that a significant minority of psychiatric inpatients has prolonged grief symptoms, more research into inpatient treatment programs is needed.

**Supplementary Information:**

The online version contains supplementary material available at 10.1186/s12888-024-05784-2.

## Background

Prolonged Grief Disorder (PGD) is a new diagnosis in both the 11th edition of the International Classification of Diseases (ICD-11; [1]) and the text revision of the 5th edition of the Diagnostic and Statistical Manual of Mental Disorders (DSM-5-TR; [2]). The core criteria of the disorder are a persistent and pervasive longing and preoccupation with the deceased for at least six months (ICD-11) or twelve months (DSM-5-TR) after the death. Accessory symptoms differ slightly between manuals and include e.g. sadness, anger, guilt in the ICD-11 and avoidance of memories, feeling alone or like one’s life no longer has meaning in the DSM-5-TR. Before its inclusion in ICD-11 and DSM-5-TR, the disorders’ name and diagnostic criteria have been subject to discussion and continued development during the last two decades [3]. Different prevalence rates were found depending on the criteria set and measure being used [4, 5]. This illustrates that prevalence- and treatment-outcome studies need to use measures that are in line with the current diagnostic guidelines. With the International Prolonged Grief Disorder Scale (IPGDS; [6]) a valid and reliable self-report questionnaire for PGD_ICD−11_ exists. However, a clinician administered interview specifically assessing PGD_ICD−11_ is still lacking [7]. We therefore developed the International Interview for Prolonged Grief Disorder according to ICD-11 (I-PGD-11). In general, self-report measures seem to find different prevalence rates of mental disorders than interview-based methods (e.g., anxiety: [8]; eating disorders: [9]; for mental disorders in children: [10]; Borderline-Personality-Disorder (BPD): [11], depression: [12]). A meta-analysis suggested that interview-based measures lead to a more valid estimation of the prevalence of mental disorders [12]. Another advantage of clinical interviews is that questions can be explained to the patient, and therefore measurement accuracy can be improved as well as missing data and non-responses can be reduced [13]. Self-report measures are time-efficient, while clinical interviews are needed for a valid diagnosis [14]. Discrepancies observed between self-report measures and clinical interviews may arise from different time frames, reminders of the instructions provided during a clinical interview, and divergent comprehension of symptoms. Reported errors, specifically general response errors during questionnaire completion, or errors in symptom attribution, have also been identified as potential reasons [14].

Around the same time we developed the I-PGD-11, the Traumatic Grief Inventory-Clinician Administered (TGI-CA; [15]) was created. It is based on the self-report questionnaire TGI-SR+ [16, 17] and allows to follow both DSM-5-TR and ICD-11 criteria for PGD. However, the authors argue in their discussion of the TGI-CA, that the wording of some items differs slightly from the criteria formulation of the manuals (e.g., regarding preoccupation with the deceased) and that the TGI-CA is a clinician administered screening instrument rather than a clinical interview that allows to make a diagnosis [15]. A clinical interview that is specifically tailored to PGD_ICD−11_ therefore constitutes a valuable addition to the diagnostic toolbox for PGD.

Our first aim of this study is to introduce the I-PGD-11 and provide first insights into its psychometric properties.

Even though PGD receives continuously increasing recognition and importance in scientific research [18], and Rosner and colleagues [19] strongly recommended healthcare providers to incorporate routine screening for PGD as an essential component of patient care it is not yet part of most standard intake assessments in psychiatric departments. The present study is therefore also aimed to shed light on the prevalence of PGD within an inpatient psychiatric setting and inform decisions to include PGD into the standard assessments.

To the best of our knowledge no study to date assessed PGD in a psychiatric inpatient sample. In representative general population and community samples, the PGD_ICD−11_ prevalence rate ranges from 1.5 to 2.4% [19–21]. The PGD_DSM−5−TR_ prevalence differs slightly and was found to be 1.5% and 1.9% in two representative German samples respectively [19, 22]. Prevalence rates for bereaved samples are naturally higher: for ICD-11 prevalence rates of 3.6–18% were found [19, 23–25]. For DSM-5-TR the prevalence rates among bereaved samples range from 3.3 to 10.1% [19, 22, 26]. Prevalence rates seem to be more similar in general population studies than in bereaved samples. In grief-treatment-seeking bereaved adults prevalence rates of 52% and 76% were found for PGD_DSM−5−TR_ and PGD_ICD−11_, respectively [27]. In Syrian refugees PGD_ICD−11_-prevalence was at 15.1% [28]. In bereaved parents ICD-11 prevalence rates ranged from 35.5% [29] to 49.5% [30]. Studies that assessed PGD after a loss of a person due to Covid-19 found prevalence rates of 37.8% [31] and 64.1% [32]. There are also specific risk factors or correlates associated with PGD which influence prevalence rates in a given sample. These can refer to both sociodemographic as well as loss related characteristics [4]. A recent meta-analysis identified several potential risk factors: female gender of the bereaved, lower educational level, death of a child or partner, unnatural, unexpected deaths, pre-loss grief or depressive symptoms and attachment anxiety as both unadjusted and adjusted predictors (i.e., associations found in univariate or multivariate analyses, respectively) of PG-symptoms [33]. Time since loss was a significant moderator and therefore also interpreted as a potential risk factor for PG-symptoms.

Focusing on PGD in a psychiatric inpatient sample is important because disordered grief is associated with a reduced quality of life, functional impairments, sleeping and substance use disorders, as well as a higher risk for heart diseases, cancer and suicidality [34, 35]. Suicidality and substance use disorders are often reasons for admission to psychiatric inpatient units [36, 37], raising the question of possibly high comorbidity with PGD. The few studies that compare inpatient and outpatient samples indicate a higher symptom load in inpatient populations in general [38, 39]. Research into disorders related to PGD suggests a higher symptom severity in inpatients as compared to outpatients. For example, the prevalence of Posttraumatic Stress Disorder (PTSD) in inpatient samples exceeded prevalence rates found in general population samples [40, 41]. Additionally, comorbidity can complicate adaptation to a loss [42] which puts people with a pre-existing mental disorder at higher risk for the development of PGD. Loss can also increase vulnerability for other (comorbid) mental disorders. This phenomenon is akin to the commonly assumed relationship in PTSD, where the diagnosis increases the likelihood of developing additional mental disorders [40]. Consequently, a pre-existing loss or even PGD itself may heighten the vulnerability of patients to develop another mental disorder that may then lead to being admitted for inpatient care.

### Aims

With the present study we first sought to evaluate the psychometric properties of the I-PGD-11 in an inpatient psychiatric sample. Secondly, the present study aimed to determine the prevalence rate of PGD in that inpatient population according to ICD-11 and DSM-5-TR. Based on evidence from studies with other inpatient samples we expect the prevalence rate to exceed rates in general population samples [19, 22, 24]. To provide a thorough and valid PGD assessment, we used a combination of self-report and clinician-administered instruments, including the newly developed I-PGD-11.

## Methods

### Design and sampling

Data collection was carried out from May 2022 until March 2023 in the open psychiatric wards and the day hospital of the hospital in Höchst, Frankfurt Main, Germany^1^. All patients who reported the loss of a close person during the course of their life were eligible to participate. Patients were excluded from the study if the severity of their psychiatric symptoms rendered it impossible to conduct clinical interviews (e.g., high level of confusion due to dementia or very high level of current psychotic symptoms).

To determine interrater reliability for the I-PGD-11, an additional sample of five patients who reported the loss of a close person was recruited from the outpatient Centre for Psychotherapy of the Goethe-University Frankfurt.

### Procedure

Approval was obtained from the ethics committee of the of the Goethe University Frankfurt (2021-62, 2023-17) and the Chamber of Hessian Physicians (2021-2730-evBO). The study was preregistered (10.17605/OSF.IO/K98MF).

**Fig. 1:**
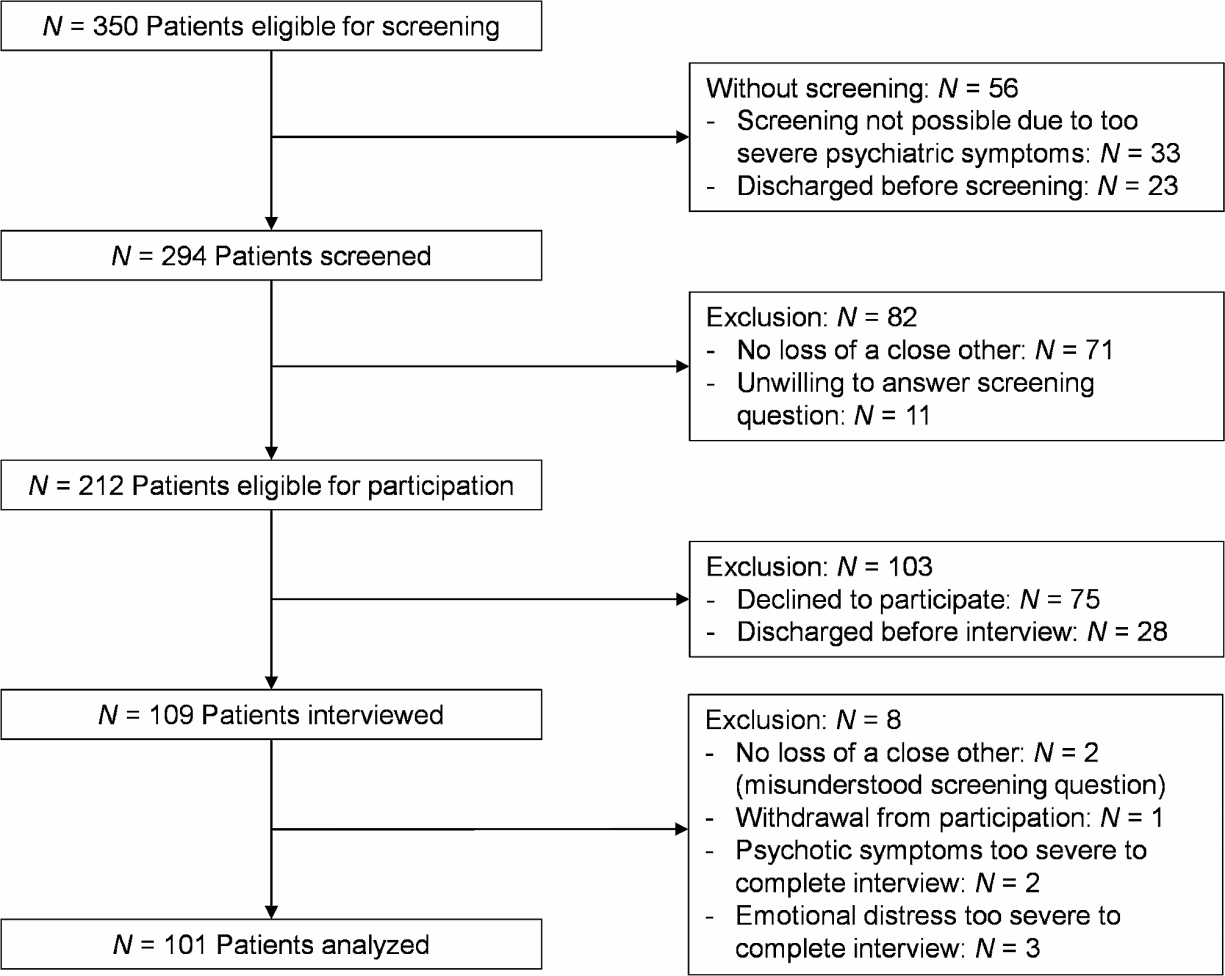
Participant flow

Upon admission, patients were screened for the death of a close person at some point in their lives (Fig. 1). Due to illness-related staff shortage, physicians were not able to screen all admitted patients and the first author conducted screenings in the last four months of the study.

Patients who had experienced a loss, were informed about the study procedure, content, and duration verbally and in writing.

Patients who gave informed consent were assessed with semi-structured interviews and questionnaires. Clinical interviews were administered first, and patients then received standardized instructions and explanations for the questionnaires. Patients who were not able to complete the interviews and questionnaires in one session, received instructions about completing the questionnaires in their own time or were invited to a second session. The complete assessment took about 90 min. The first author (MSR), a trained clinician rater, conducted the clinical interviews and administered the self-report instruments. Uncertainties regarding the diagnostic status were resolved in weekly meetings among MSR, FLM, and RS. The I-PGD-11 and Prolonged Grief 13 Revised (PG-13-R; 18) was also administered to the five patients of the outpatient sample. These interviews were videotaped and rated by another trained rater.

### Measures

Clinician-rated PGD_ICD−11_ was assessed with the German version of the I-PGD-11 (see Additional File 1 and Additional File 2 for the English translation). The newly developed interview consists of 13 questions with a 5-point Likert response format (1 = Not at all to 5 = Overwhelmingly) which follows the PG-13-R [18]. One additional question inquires about the person who died, and two open questions assess the culturally normative grief period and reaction (cultural caveat) and cause of death, respectively. Impairments caused by the grief symptoms are rated in a binary format (Yes vs. No). Items were constructed to reflect the wording of the ICD-11 criteria for PGD. The I-PGD-11 allows to assess both the diagnostic status and the severity of PGD_ICD−11_ symptoms. The diagnostic status can be evaluated with a strict or with a moderate diagnostic algorithm [6, 24]. Strict criteria require that one of the items 2 or 3, and at least one of the items 4–14 are all rated with 4 or 5, and the impairment (PGD_18) and cultural-caveat-items (PGD_15 to _17) are affirmed. For the moderate diagnostic algorithm, all items with a Likert response format must be rated 3 or higher. The severity of the PGD_ICD11_-symptoms is calculated as the sum score of the 13 items with a Likert response format (PGD_2 through PGD_14). We assume the Likert response format to represent equidistant categories with an underlying continuum. PGD_DSM−5−TR_ was assessed with the PG-13-R [18], which had a good internal consistency (α = 0.90, ω = 0.92) and an excellent interrater reliability (ICC = 0.995, 95%-CI = 0.991–0.997, *p* < 0.01) in this sample. Other psychiatric diagnoses were made according to ICD-10 using the Mini Diagnostic Interview for psychiatric disorders Open Access [43, 44]. It is a short structured clinical interview and has good psychometric properties [45]. Self-reported PGD_ICD−11_-symptoms were assessed with the IPGDS [6] which had an excellent internal consistency in this sample (α = 0.92, ω = 0.94). Other psychological and physical symptoms were assessed with the Brief Symptom Inventory (BSI; [46]). It measures 53 symptoms with a response format from 0 (= not at all) to 4 (= extremely) and had an excellent internal consistency in this sample (α = 0.96, ω = 0.97).

### Data analysis

#### Psychometric properties of the I-PGD-11

Data were analyzed using R version 4.3.1 [47]. To inspect dimensionality of the I-PGD-11, we first performed an Exploratory Factor Analysis (EFA) of the items with a Likert response format of the interview using the R-packages corrplot version 0.92 [48], psych version 2.3.6 [49] and GPArotation version 2023.3-1 [50]. We determined the number of factors in three ways. First, we used the Kaiser-Guttman-Criterion (eigenvalue > 1; [51]) calculated with the package FactoMineR version 2.8 [52], then we assessed the scree plot and last, we used parallel analysis. We extracted the eigenvalues using a Principal Component Analysis (PCA). We used Principal Axis Factoring as the extraction method and rotated the extracted factors with the oblique (“oblimin”) rotation method. We then inspected the factor loadings. Item analysis was performed to determine the item difficulty and discrimination as indicators of item quality of the single items with the R-package sjPlot version 2.8.14 [53]. We compared the distribution of I-PGD-11 sum scores to a normal distribution graphically using ggplot2’s version 3.5.0 extension ggh4 × [54] and with the Shapiro-Wilk normality test.

We inspected reliability through internal consistency and the interrater reliability of the I-PGD-11. Internal consistency was determined with Cronbach’s Alpha and McDonald’s Omega using the psych package version 2.3.6 [49]. For Mc Donald’s Omega, values over 0.70 are interpreted as an acceptable internal consistency [55]. For the interrater reliability, we used the Intra Class Correlation (ICC) as calculated with the package irr version 0.84.1 [56].

The validity of the I-PGD-11 was inspected through concurrent-, known-groups-, and discriminant validity. The concurrent validity was evaluated by calculating the concordance of the sum scores of the I-PGD-11 with the IPGDS and the PG-13-R scores using Spearman Rank correlations since the assumption of normality for Pearson correlations was violated. To test the known-groups validity we compared I-PGD-11 sum scores and prevalence rates in known predictors of PGD with the Wilcoxon test: gender (women vs. men), educational level (more vs. less than 12 years of school), kinship to the deceased (loss of child/ spouse vs. other), time since loss (in years), cause of death (natural vs. unnatural death), and expectedness of the loss (expected vs. surprising). The discriminant validity was calculated by assessing the concordance of the I-PGD-11 with the sum score of the BSI and its subscale-values. The BSI measures diverse psychological symptoms such as hostility and psychoticism that are not closely related to grief. It allows us to evaluate whether the I-PGD-11 assesses PGD-related symptoms specifically, which can be differentiated from general psychological distress.

We used receiver operating characteristics (ROC) analysis to determine (1) the provisional cut-off-scores for a PGD-diagnosis according to the strict diagnostic algorithm when evaluating diagnostic status in the I-PGD-11 and (2) the number of accessory symptoms (C-criterion) that best distinguishes between people with and without the strict PGD-diagnosis according to I-PGD-11. To perform the ROC-analyses in R we used the packages pROC version 1.18.4 [57] and randomForest version 4.7–1.1 [58]. The Youden-Index yielded the results for the cut-off-score and the number of accessory symptoms with a good match of high sensitivity and specificity.

#### Prevalence rates

To investigate the prevalence of PGD_ICD−11_, we first evaluated the diagnostic statuses among all interviewed patients according to the I-PGD-11 and the IPGDS using strict and moderate criteria for PGD. For a diagnosis of PGD_DSM−5−TR_, one of the items 3 or 4 and at least three of the items 5–12 of the PG-13-R must be rated 4 or higher, while item 13 must be affirmed. We also calculated the agreement between the I-PGD-11, PG-13-R and IPGDS and diagnostic algorithms using Cohen’s kappa.

#### Missing values

We excluded one patient from the analyses of the psychometric properties of the I-PGD-11 and PG-13-R due to missing values on those measures. However, for the calculation of prevalence rates, their data were retained based on the existing answers. Five patients did not complete the IPGDS, they were excluded from the analyses concerning the IPGDS. Five other patients had one missing value on the IPGDS each. They were included in the calculation of prevalence rates based on the IPGDS, but we used pairwise exclusion in other analyses involving the IPGDS. Six patients did not fill out the BSI and were excluded from the respective analyses. Cases with single missing values on the BSI were handled as suggested in the instrument’s manual.

## Results

### Sample characteristics

Sociodemographic and loss-related characteristics of the sample are presented in Table 1. Participants were on average 45.27 years old (*SD* = 14.66, *range*: 18–86 years). Forty-six (*n* = 45.54%) participants identified as female, 54 as male (*n* = 53.47%) and one as non-binary (*n* = 0.99%). A migration background was reported by 46.53% of patients (Turkey: 10.89%, Poland: 6.93%, Croatia/ Philippines/ Russia: each 2.97%, other countries: 19.8%). Two patients reported grief as the reason for in-patient treatment; all other patients were admitted for other reasons. Drug or alcohol abuse or detox were stated most often as a reason for admission (28.48% of reasons), followed by depression (23.18% of reasons), suicidality (10.61%), anxiety (9.27%) and other reasons (28.48%). Patients met a mean of 2.53 comorbid diagnoses (*range*: 1–8), while most patients had one or three diagnoses (28.71%, respectively). Most participants had lost other family members (e.g., siblings, grandparents, etc.) or friends (48.51%). The mean time since loss was 11.06 years (*SD* = 11.19, *range*: 0.02–53.00 years). Most deaths were natural (79%), but unexpected (61.39%).

**Table 1:** Sociodemographic and loss-related characteristics

Characteristic	Total sample (*N* = 101)
Gender	
Female, n (%) Male, n (%) Non-binary, n (%)	46 (45.54%) 54 (53.47%) 1 (0.99%)
Age in years, M (SD)	45.27 (14.66)
Migration background, n (%)	47 (46.53%)
Marital status, n (%)	
Single Married In a partnership, but unmarried Living apart, not yet divorced Divorced Widowed	55 (54.46%) 19 (18.81%) 5 (4.95%) 1 (0.99%) 15 (14.85%) 6 (5.94%)
Educational Background, n (%)	
Still in school Special education school^1^ Graduation after 9 years of school^2^ Graduation after 10 years of school^3^ Graduation after 12/13 years of school^4^ Left school without graduating	1 (0.99%) 1 (0.99%) 15 (14.85%) 30 (29.70%) 45 (44.55%) 9 (8.91%)
Employment, n (%)	
Employed Unemployed Retired Other	33 (32.67%) 32 (31.68%) 19 (18.81%) 17 (16.83%)
Psychiatric diagnoses according to the Mini-DIPS-OA^6^, ICD-10, n (%) Any mental disorder due to brain damage (F06.0 – F06.9) Any substance-use-disorder (F10.0 – F19.9) Any psychotic disorder (F20.0 – F29.9) Any bipolar disorder (F31.0 – F31.9) Any depressive disorder (F32.0 – F39.9) Any anxiety disorder (F40.0 – F41.9) Any obsessive-compulsive disorder (F42.0 – F42.9) Acute Stress Disorder (F43.0) Posttraumatic Stress Disorder (F43.1) Any somatoform disorder (F45.0 – F45.9) Any eating disorder (F50.0 – F50.9) Any sleep disorder (F51.0 – F51.9) Any postnatal mental disorder (F53.0 – F53.9) Any impulse control disorder (F63.0)	1 (0.99) 59 (58.41) 11 (10.89) 8 (7.92) 59 (58.41) 37 (36.63) 2 (1.98) 1 (0.99) 23 (22.77) 9 (8.91) 3 (2.97) 9 (8.91) 1 (0.99) 3 (2.97)
Person who died, n (%)	
Child Partner Parent Other	3 (2.97%) 10 (9.90%) 39 (38.61%) 49 (48.51%)
Cause of death, n (%)^5^	
Natural Unnatural	79 (79.00%) 21 (21.00%)
Expectation of the death, n (%)	
Expected Unexpected	39 (38.61%) 62 (61.39%)
Time since loss in years, M (SD)	11.06 (11.19)
1 – German “Förderschule” 2 – German “Hauptschule” 3 – German “Realschule” 4 – German “Abitur” 5 – Cause of Death refers to *N* = 100 patients, 1 missing value 6 – Mini Diagnostic Interview for psychiatric disorders Open Access; personality disorders are not assessed by the Mini-DIPS-OA; Patients reported M = 2.53 diagnoses (range 1–8), therefore multiple diagnoses per patient are possible; only one diagnosis of the category is counted per patient, but patients sometimes had more than one diagnosis in that category (e.g., F10.0-F19.9)	

### Psychometric properties of the I-PGD-11

**Fig. 2:**
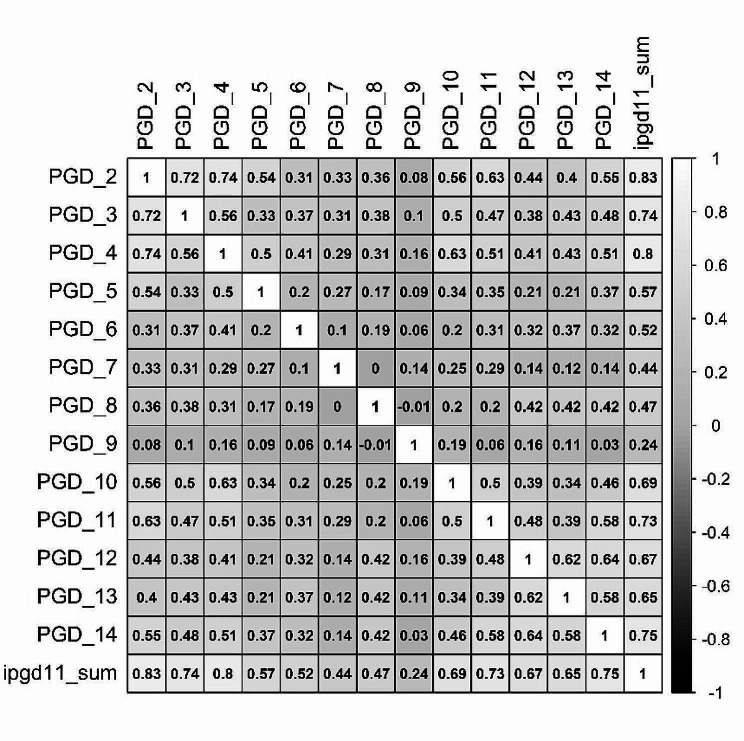
Correlation matrix of the I-PGD-11

**Fig. 3:**
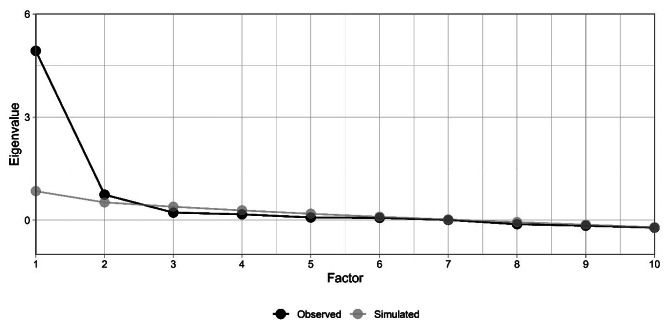
Scree plot

**Fig. 4:**
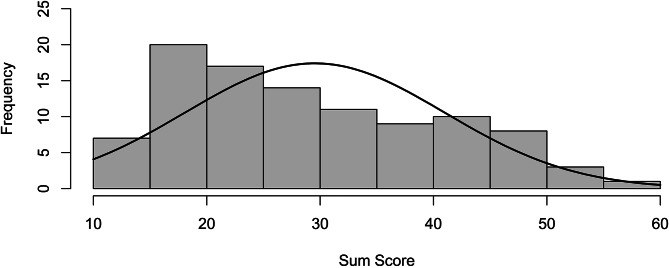
Test score analysis

Correlations among the 13 I-PGD-11 items with a Likert response format and the sum score are depicted in Fig. 2, and item mean values, standard deviations, skewness, difficulty, and discrimination are depicted in Table 2. The EFA yielded three factors with eigenvalues greater than 1.0 (i.e., 5.45, 1.40, 1.03; see Table 3). Figure 3 depicts the scree plot including the parallel analysis. In the parallel analysis two factors were above the simulated curve, which pointed towards a two-factor solution. However, the graph clearly depicts an “elbow” after the first factor. Therefore, a one-factor solution could also be plausible. Taken together, the three methods to determine the number of factors did not yield uniform results. We discarded the three-factor solution according to the Kaiser-Criterion since it seems to overestimate the number of factors [59]. When calculating the parallel analysis, we mostly received the result of a two-factor solution but depending on the seed, number of iterations and factor method, some results also pointed towards a one-factor solution. Given that the factors other than the first factor did only explain little variance and generally, more sparse factor solutions are recommended, we opted for a one-factor solution. The factor loadings are depicted in Table 2. Most items loaded above 0.5 on this one factor which can be seen as support for the decision for the one-factor-solution. Item 9 had a very low loading on the factor and a closer inspection revealed that its formulation was too narrow. The distribution of sum scores of the I-PGD-11 including all items with a Likert response format as compared to a normal distribution are depicted in Fig. 4. I-PGD-11 sum scores were not normally distributed in the current sample (Shapiro-Wilk normality test: *W* = 0.95, *p* < 0.01, and Fig. 4).

The internal consistency was good (α = 0.89 and ω = 0.92). Interrater reliability was excellent (ICC = 0.985, 95%-CI = 0.977–0.991, *p* < 0.01). The concurrent validity between I-PGD-11 and IPGDS and PG-13-R were high (*ρ* = 0.86, *p* < 0.01 and *ρ* = 0.90, *p* < 0.01, respectively). I-PGD-11 sum scores were significantly higher for women (vs. men, see Table 4). Patients with less than 12 years of schooling had significantly higher sum scores than patients with 12 years of schooling or more. Patients who had lost a child or spouse had significantly higher sum scores than patients with a loss of another person close to them. Unnatural and surprising deaths were associated with significantly higher sum scores. The association between time since loss and sum score was not significant. There was medium concordance between the I-PGD-11 sum score and the BSI sum score (*ρ* = 0.54, *p* < 0.01) and medium correlations with the BSI subscales (somatization: *ρ* = 0.45, *p* < 0.01; obsession-compulsion: *ρ* = 0.41, *p* < 0.01; interpersonal sensitivity: *ρ* = 0.36, *p* < 0.01; depression: *ρ* = 0.43, *p* < 0.01; anxiety: *ρ* = 0.46, *p* < 0.01; hostility: *ρ* = 0.31, *p* < 0.01; phobic anxiety: *ρ* = 0.46, *p* < 0.01; paranoid ideation: *ρ* = 0.42, *p* < 0.01; psychoticism: *ρ* = 0.43, *p* < 0.01).

**Fig. 5:**
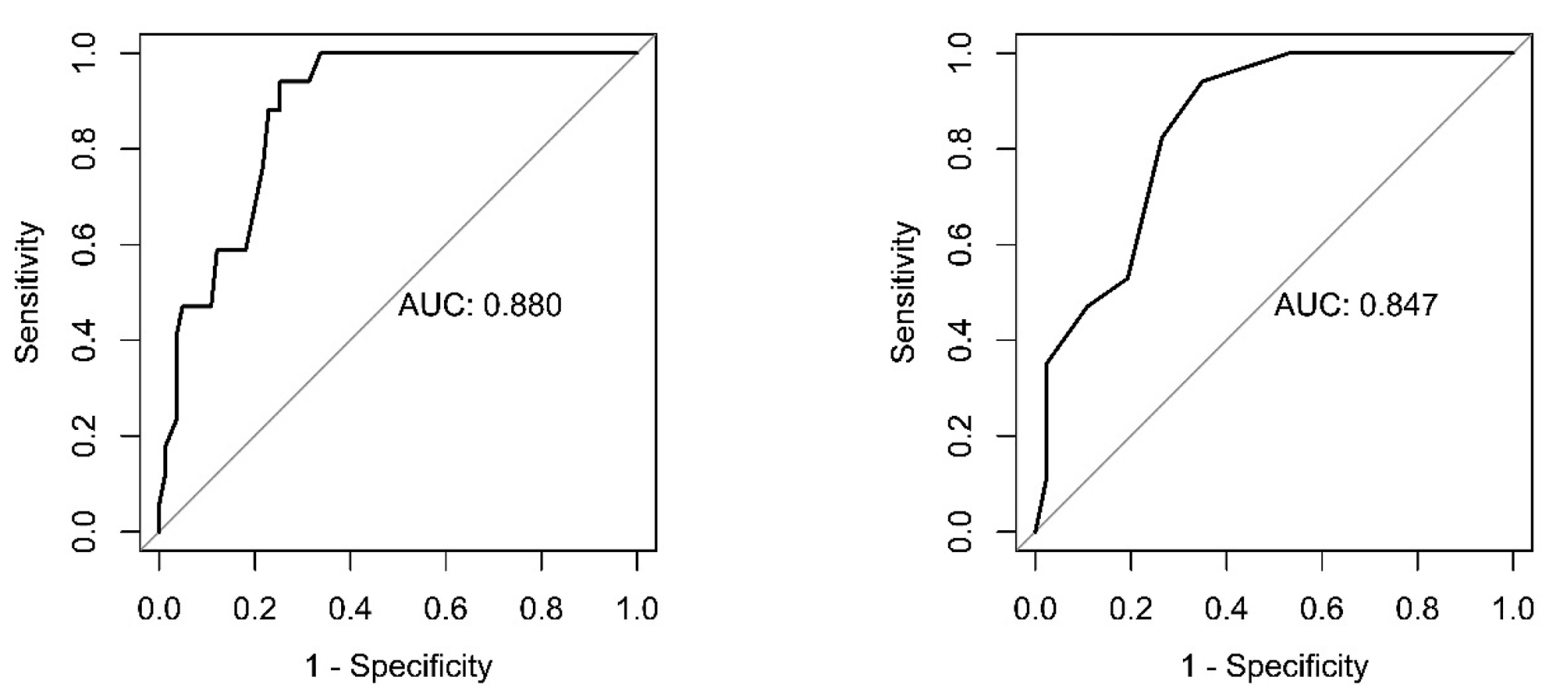
**a**. ROC-analysis of the I-PGD-11 Cutoff Score. **b**. ROC-analysis of the accessory symptoms

The Youden-Index in the ROC-analysis yielded a provisional cut-off score (see Fig. 5a) of 32.5 (13 items with Likert response format, range 13–65) with a sensitivity of 0.941, specificity of 0.747, and an AUC of 0.88 (95% CI: 0.81–0.94). The Youden-Index in the ROC-analysis identified that having 2.5 accessory symptoms yielded a sensitivity of 0.941 and a specificity of 0.651 for distinguishing diagnosis fulfillment. The AUC was 0.85 (95% CI: 0.76–0.92, see Fig. 5b).

**Table 2:** I-PGD-11 Itemanalysis

Item	Mean	SD	Skew	Item Difficulty	Item Discrimination	Alpha if deleted	Factor Loadings in EFA (after oblimin factor rotation)
PGD_2	2.71	1.42	0.2	0.54	0.78	0.85	0.84
PGD_3	2.67	1.38	0.31	0.53	0.68	0.85	0.72
PGD_4	2.98	1.52	0	0.60	0.74	0.85	0.79
PGD_5	3.46	1.35	-0.46	0.69	0.48	0.87	0.51
PGD_6	2.1	1.53	0.97	0.42	0.41	0.87	0.45
PGD_7	2.38	1.62	0.64	0.48	0.31	0.88	0.33
PGD_8	1.46	1.08	2.26	0.29	0.39	0.87	0.45
PGD_9	1.45	1.06	2.3	0.29	0.15	0.88	0.15
PGD_10	2.24	1.51	0.76	0.45	0.61	0.86	0.65
PGD_11	2.71	1.64	0.21	0.54	0.65	0.86	0.70
PGD_12	1.69	1.19	1.63	0.34	0.61	0.86	0.65
PGD_13	1.71	1.33	1.61	0.34	0.58	0.86	0.62
PGD_14	1.97	1.47	1.17	0.39	0.68	0.85	0.75

Mean inter-item-correlation = 0.339, Cronbach’s alpha = 0.872

**Table 3:** Eigenvalues and Explained Variance

Factor	Eigenvalue	Explained Variance	Cum. Explained Variance
1	5.45	41.91	41.91
2	1.40	10.74	52.64
3	1.03	7.92	60.56
4	0.83	6.42	66.99
5	0.79	6.06	73.04
6	0.75	5.79	78.83
7	0.69	5.29	84.11
8	0.49	3.80	87.91
9	0.44	3.38	91.30
10	0.35	2.67	93.96
11	0.33	2.54	96.50
12	0.30	2.32	98.82
13	0.15	1.18	100.00

**Table 4:** Sociodemographic and loss-related correlates of I-PGD-sum-scores (*N* = 100)

	I-PGD-11 Sum Score Mean (Range)	Test statistic^1^
Gender		
Men Women	27.21 (13–52) 32.19 (16–58)	*W* = 1542 *p* = 0.01
Educational Level		
School for < 12 years School for ≥ 12 years	33.15 (13–52) 25.11 (13–58)	W = 741 *p* < 0.001
Kinship to the deceased		
Loss of child/ spouse Other	37.67 (19–58) 28.42 (13–52)	*W* = 751 *p* = 0.02
Time since loss (in years)^2^		*ρ*= -0.08 *p* = 0.43
Cause of death		
Natural Unnatural	27.92 (13–52) 36.35 (17–58)	*W* = 480 *p* < 0.01
Expectedness of the loss		
Expected Surprising	25.67 (13–52) 32.00 (13–58)	*W* = 801.5 *p* < 0.01

^1^ Test statistic refers to Wilcoxon Test

^2^ Test statistic for Time since loss: Spearman Rank Correlation

### Prevalence rates

Conditional prevalence rates are presented in Table 5. The prevalence rate of PGD_ICD−11_ among bereaved patients as assessed with the I-PGD-11 was 16.83% when using the strict diagnostic algorithm and 22.77% when using the moderate diagnostic algorithm. Using self-reported grief symptoms on the IPGDS yielded slightly lower prevalence rates of PGD_ICD−11_ (10.41% using the strict diagnostic algorithm and 20.83% using the moderate diagnostic algorithm). Prevalence of PGD_DSM−5−TR_ was 10.89%.

The agreements between the different diagnoses (ICD-11 vs. DSM-5-TR) and diagnostic algorithms (strict vs. moderate) are depicted in Table 6. The highest concordance was between I-PGD-11 (strict) and PG-13-R (κ = 0.67, substantial), the lowest between IPGDS (strict) and PG-13-R (κ = 0.33, fair).

**Table 5:** Prevalence rates of Prolonged Grief Disorder according to ICD-11 and DSM-5-TR

	I-PGD-11 (strict, *N* = 101)	I-PGD-11 (moderate, *N* = 101)	IPGDS (strict, *N* = 96)	IPGDS (moderate, *N* = 96)	PG-13-R (*N* = 101)
Prevalence Rates, % (n)	16.83% (17)	22.77% (23)	10.41% (10)	20.83% (20)	10.89% (11)

I-PGD-11 = International Interview for Prolonged Grief Disorder according to ICD-11

IPGDS = International Prolonged Grief Disorder Scale

PG-13-R = Prolonged Grief 13 Revised

Strict = strict diagnostic algorithm, moderate = moderate diagnostic algorithm

**Table 6:** Concordance between PGD_ICD−11_ (strict and moderate diagnostic algorithm, I-PGD-11 and IPGDS) and PGD_DSM−5−TR_ examined with Cohen’s Kappa

	I-PGD-11 (strict, *N* = 101)	I-PGD-11 (moderate, *N* = 101)	IPGDS (strict, *N* = 96)	IPGDS (moderate, *N* = 96)	PG-13-R (*N* = 101)
I-PGD-11 (strict)	*κ* = 1.00	*κ* = 0.81	*κ* = 0.38	*κ* = 0.52	*κ* = 0.67
I-PGD-11 (moderate)	*κ* = 0.81	*κ* = 1.00	*κ* = 0.51	*κ* = 0.66	*κ* = 0.52
IPGDS (strict)	*κ* = 0.38	*κ* = 0.51	*κ* = 1.00	*κ* = 0.54	*κ* = 0.33
IPGDS (moderate)	*κ* = 0.52	*κ* = 0.66	*κ* = 0.54	*κ* = 1.00	*κ* = 0.38
PG-13-R	*κ* = 0.67	*κ* = 0.52	*κ* = 0.33	*κ* = 0.38	*κ* = 1.00

I-PGD-11 = International Interview for Prolonged Grief Disorder according to ICD-11

IPGDS = International Prolonged Grief Disorder Scale

PG-13-R = Prolonged Grief 13 Revised

Strict = strict diagnostic algorithm

Moderate = moderate diagnostic algorithm

κ = Cohen’s Kappa (< 0.00 = poor, 0–0.20 = slight, 0.21–0.40 = fair, 0.41–0.60 = moderate, 0.61–0.80 = substantial, 0.81–1.00 = almost perfect agreement)

## Discussion

The present cross-sectional study investigated the psychometric properties of a new clinical interview for PGD_ICD−11_ and reports preliminary prevalence of PGD according to ICD-11 and DSM-5-TR in an inpatient psychiatric sample. PGD was assessed with two clinical interviews and one self-report questionnaire. Psychiatric inpatients are a sample rarely included in studies and have not yet been examined regarding PGD.

The psychometric properties of the newly introduced I-PGD-11 suggest very good performance characteristics. The I-PGD-11 therefore appears to be a reliable and valid instrument to diagnose PGD according to the ICD-11. Furthermore, it can be used in highly burdened samples of psychiatric inpatients as it was first examined in this population. The only adjustment that was needed after the psychometric evaluation was to item 9. We adjusted item 9 from “Do you feel like the person you’ve lost is to blame for their death?” to “Do you feel like the person you’ve lost or other people are to blame for their death?” (see Additional File 1 for the final German version and Additional File 2 for the final English translation). This adjustment was done because its original wording was too narrow and the item had a low loading on the factor for the one-factor solution. We then extended this formulation to other persons since this is more in line with previous suggestions regarding the interpretation of the ICD-11-formulation of “blame” [7]. The factor analysis suggested one factor for the I-PGD-11. This is in line with existing evidence for a uniform structure of PGD symptomatology because a one-factor solution was also found in the IPGDS [6], the PG-13-R [18] and the TGI-CA [15]. Our results regarding convergent, known-groups and discriminant validity demonstrate the strong performance of the I-PGD-11, confirming its ability to effectively capture PGD and differentiate it from other constructs like hostility and psychoticism. Patients with high I-PGD-11 scores also had high PG-13-R and IPGDS-scores, which means that convergent validity is high between those established instruments. Female gender, lower educational level, kinship to the deceased, as well as unnatural and unexpected deaths were associated with higher PGD scores according to the I-PDG-11 which is in line with a recent meta-analysis [33]. Unlike in previous studies (e.g., [15]) and this meta-analysis, time since loss did not predict higher PGD symptom severity. However, this does not necessarily mean, that time since loss is irrelevant as a risk factor for PGD as the meta-analysis mainly identified it as a moderator when PGD-symptoms after the death of a partner were examined [33]. It rather highlights that time since loss is not a universal risk factor, that patients who experienced bereavement a long time ago might still meet the diagnostic criteria, and more research is needed on potential moderators for these risk factors.

Criteria for PGD have undergone important changes during their development (e.g., [3]) and there is still some controversy regarding the necessity of the diagnosis [60] but also the specific criteria that should be included [7]. With the high prevalence rate in the current sample and the replication of a one-factor solution of PGD, the present study lends more evidence to the importance of PGD as a stand-alone uniform syndrome. In addition, the number of accessory symptoms needed for a diagnosis is criticized as too low [7]. The results of our ROC-analysis suggest three accessory symptoms to be ideal for a PGD diagnosis. This is in line with the TGI-CA, where the authors found the highest agreement between ICD-11 and DSM-5-TR diagnosis when using three accessory symptoms for PGD_ICD−11_ [15]. However, in a grief-treatment-seeking sample six accessory symptoms were found to be the ideal number for a PGD_ICD−11_-diagnosis [61]. In another study four accessory symptoms yielded the highest agreement between PGD_ICD−11_- and PGD_DSM−5−TR_-diagnoses [19]. These differences could be due to several factors, e.g., sample characteristics or the different measures used. There is more research needed to determine the optimal number of accessory symptoms for the PGD_ICD−11_-diagnosis, e.g., in meta-analyses or studies to determine potential moderators like sample characteristics which influence the optimal number of accessory symptoms found in the studies (see also [62]).

The agreement between the different diagnoses or assessment types was lower than expected. There might be several reasons for that. Differences in item-wording might have led to the discrepancies because patients might have confirmed still feeling intense longing or yearning, as phrased in the PG-13-R, but might not experience this as persistent and pervasive, as phrased in the I-PGD-11. Patients frequently did not meet the strict PGD diagnosic criteria in their IPGDS responses, often due to selecting “3 - Sometimes” for the impairment or cultural caveat items. The same response possibly led to rating these criteria as fulfilled on the binary item format of the I-PGD-11, thus creating a discrepancy. Additionally, while the response format in I-PGD-11 and PG-13-R was an identical agreement response format, the IPGDS uses a different frequency response format. In a study that investigated the effect of those response formats when assessing job stress, the authors found slightly higher scores when using an agreement response format [63]. The same effect might be applicable when comparing the diagnostic status or sum scores of I-PGD-11 or PG-13-R with the IPGDS. Overall, these findings highlight the importance of carefully considering the wording and response format of assessment tools to ensure accurate and consistent assessment of PGD. Further research is needed to refine and standardize assessment methods to improve agreement across different diagnostic measures.

The prevalence rate of PGD_ICD−11_ was 16.83% according to the I-PGD-11 and 10.41% according to the IPGDS (using the strict diagnostic algorithm). Prevalence of PGD_DSM−5−TR_ was at 10.89%. Therefore, the prevalence rates found in our study exceed prevalence rates found in general population studies of bereaved people (PGD_ICD-11_: 4.2%, PGD_DSM-5-TR_: 3.3%, [19]; PGD_ICD-11_: 6.9–12.6%, [24]; pooled PGD-prevalence: 9.8%, [64]). However, the prevalence rates resemble those in other specific bereaved subgroups. While the prevalence rates in refugees were comparable (PGD_ICD-11_: 15.1%, [28]), prevalence rates among bereaved parents (PGD_ICD-11_: 49.5%, [30]) or people bereaved to Covid-19 (PGD_DSM-5-TR_: 64.1%, [32]) seem to be higher. This underlines the influence of certain characteristics of the loss as risk factors for a manifestation of PGD. Losing a child or losing someone unexpectedly for example puts someone more at risk for PGD [33], which would be the case in bereaved parents or people bereaved to Covid-19. However, these risk factors were mixed in the present sample, which could explain why the prevalence rate is higher than in general population studies and lower than in studies of samples with specific risk factors.

To the best of our knowledge, PGD is not yet part of the standard assessments in the health care system, especially in psychiatric wards, although our findings highlight the necessity. There might be concerns towards asking patients whether they have lost someone close to them similar to the barriers in mental health care staff to ask patients about aversive or potential traumatic experiences. Reasons are often fear of increasing distress, not being able to cope with responses themselves, more urgent concerns and lacking knowledge on the way to ask and respond to traumatic experiences [65]. As a result, PTSD is often overlooked even during inpatient stays [40, 41]. Additionally, there can be organizational factors keeping staff from asking about traumatic experiences, e.g., low morale or a lacking team-culture to do so [66]. Similar barriers could be in process when addressing loss and grief in general but especially in inpatients, where distress and symptom severity are already high. A study with people bereaved by suicide or under other traumatic circumstances suggested that people might feel that they can only talk about grief and loss if they are directly asked about it [67]. In their analysis on grief and mental health, Young et al. [68] hint that grief and loss are often not addressed in psychiatric care. However, asking for grief and loss in a psychiatric setting presents a great benefit because it allows to choose the best treatment available for the patient. For example, antidepressant medication has no effect on PGD-symptoms [3, 69] and a diagnosis of PGD is a first step to adding additional treatment components. Existing psychological treatments have proven to be highly specific to grief in inpatient settings, emphasizing the need for those intervention additions [70].

**Strengths and limitations.** A major strength of our study is our population, which is very rarely investigated. Despite the substantial effort required to recruit this population, the sample is diverse regarding age, gender, migration background, and reason for admission. In addition, we used three different measures to assess PGD thereby allowing a comparison between clinician-administered and self-report measure as well as ICD-11 and DSM-5-TR. We also followed the most recent criteria of PGD. For PGD_ICD−11_ we used our new I-PGD-11, which closely followed the formulation of the criteria. By using other new measures for PGD, we also contribute to investigations into their reliability and validity. The IPGDS and the PG-13-R have been investigated before, but the present study further proved their applicability in highly distressed samples like our inpatient population.

There are also some limitations that should be considered. First, we interviewed patients exclusively from one psychiatric department in a single hospital, limiting the generalizability of our findings. Thus, the sample was not representative. In 2022, German psychiatric inpatients averaged 45 years in age, with 51% male and 49% female [71]. They were primarily treated for mood, neurotic, stress-related, somatoform, and sleep disorders (18.4%), followed by mental and behavioral disorders due to psychoactive substance use (11.3%). While our sample shares a similar gender and age distribution, it had a higher prevalence of admissions for substance use disorders. Second, in the beginning of the study there was no extensive screening of all admitted patients. We adjusted the screening procedure in a way that the first author was responsible for screening, but for the first seven months some relevant data (patients admitted, patients screened and their responses) are missing. Despite repeated reminders, doctors did not universally screen all patients, suggesting potential structural (e.g., time constraints) or personal (e.g., reluctance to inquire about grief and loss) barriers to screening. Third, we used a new interview to diagnose PGD_ICD−11_ which was not examined in an independent sample before. When we started our study there was no clinician-administered interview available for the assessment of PGD_ICD−11_. To evaluate reliability, we only used internal consistency and interrater reliability. It should be noted, that internal consistency is often critiqued as not sufficiently depicting reliability [72] or rather only depicting data quality [73]. Therefore, more research is needed to investigate the reliability of the I-PGD-11, more specifically e.g., retest-reliability. Inspection of discriminant validity was also limited to instruments assessing symptoms of other mental health disorders and future studies should also investigate associations with conceptually different constructs (e.g., well-being). Additionally, the psychometric properties only apply to the German version of the I-PGD-11. The I-PGD-11 was translated to English by our workgroup and then reviewed by a professional English and German interpreter. The response format’s translation was adopted from the German translation of the PG-13-R [18] since our response format was designed to be identical. Future research needs to evaluate the psychometric properties of the English translation. While the choice of our sample yields valuable insights into PGD in psychiatric inpatients, one must bear in mind that this is a highly distressed population. The patients were often experiencing acute symptoms or were under the influence of medication which might have influenced their understanding of items. Therefore, we had to be flexible in our procedure with the questionnaires and could not keep the procedure identical in all patients. Because of the cross-sectional study design, we cannot infer from our results whether the loss of a close other leads to a higher probability of developing other mental disorders or whether already existing mental disorders heighten the probability of a PGD-onset after losing a close other. Future studies should address this with longitudinal designs.

## Conclusion

In conclusion, the I-PGD-11 is a valid and reliable measure to assess PGD. Our study of PGD prevalence rates among psychiatric inpatients underscores the importance of acknowledging this disorder within psychiatric care. We hope to raise awareness about PGD in psychiatric inpatients among both inpatient and outpatient staff, as well as the public. We further hope that the present study underlines the significance of inquiring about grief and loss in the assessment of patients with various mental disorders.

### Electronic supplementary material

Below is the link to the electronic supplementary material.

Supplementary Material 1

Supplementary Material 2

## Data Availability

The datasets generated and analysed during the current study are not publicly available due to the high sensitivity of data from psychiatric inpatients but are available from the corresponding author on reasonable request. An R-Script of our analysis as well as the codebook can be found in our preregistration on the Open Science Framework (https://doi.org/10.17605/OSF.IO/K98MF).

## Footnotes

1 varisano Klinikum Frankfurt Höchst, Klinik für psychische Gesundheit.

## Abbreviations

PGD: Prolonged Grief Disorder
I-PGD-11: International Interview for Prolonged Grief Disorder according to the ICD-11
ICD-11: International Classification of Diseases – 11th edition
DSM-5-TR: Diagnostic and Statistical Manual of Mental Disorders – 5th edition – text revision
PTSD: Posttraumatic Stress Disorder
TGI-CA: Traumatic Grief Inventory – Clinician Administered
PG-13-R: Prolonged Grief 13 Revised
IPGDS: International Prolonged Grief Disorder Scale
BSI: Brief Symptom Inventory
EFA: Exploratory Factor Analysis
ICC: Intra Class Correlation
ROC: Receiver Operator Characteristics

## References

[1] World Health Organization. ICD-11. Int Classif Dis 11th Revis. 2018.

[2] APA (2022). Diagnostic and statistical manual of mental disorders: DSM-5-TR. Fifth edition, text revision.

[3] Prigerson HG, Kakarala S, Gang J, Maciejewski PK (2021). History and status of prolonged grief disorder as a Psychiatric diagnosis. Annu Rev Clin Psychol 7 Mai.

[4] Boelen PA, Lenferink LIM (2020). Comparison of six proposed diagnostic criteria sets for disturbed grief. Psychiatry Res.

[5] Maciejewski PK, Maercker A, Boelen PA, Prigerson HG (2016). Prolonged grief disorder’ and ’persistent complex bereavement disorder’, but not ’complicated grief’, are one and the same diagnostic entity: an analysis of data from the Yale Bereavement Study. World Psychiatry Januar.

[6] Killikelly C, Zhou N, Merzhvynska M, Stelzer EM, Dotschung T, Rohner S (2020). u. a. development of the international prolonged grief disorder scale for the ICD-11: measurement of core symptoms and culture items adapted for Chinese and german-speaking samples. J Affect Disord.

[7] Eisma MC, Rosner R, Comtesse H (2020). ICD-11 prolonged grief disorder criteria: turning challenges into opportunities with multiverse analyses. Front Psychiatry Januar.

[8] Andrews B, Hejdenberg J, Wilding J (2006). Student anxiety and depression: comparison of questionnaire and interview assessments. J Affect Disord.

[9] Fairburn CG, Beglin SJ (1994). Assessment of eating disorders: interview or self-report questionnaire?. Int J Eat Disord 1 Dezember.

[10] Goodman A, Heiervang E, Fleitlich-Bilyk B, Alyahri A, Patel V, Mullick MSI (2012). u. a. cross-national differences in questionnaires do not necessarily reflect comparable differences in disorder prevalence. Soc Psychiatry Psychiatr Epidemiol 1 August.

[11] Hopwood CJ, Morey LC, Edelen MO, Shea MT, Grilo CM, Sanislow CA (2008). u. a. A comparison of interview and self-report methods for the assessment of borderline personality disorder criteria. Psychol Assess März.

[12] Lim GY, Tam WW, Lu Y, Ho CS, Zhang MW, Ho RC (2018). Prevalence of Depression in the community from 30 countries between 1994 and 2014. Sci Rep 12 Februar.

[13] Marta-Pedroso C, Freitas H, Domingos T (2007). Testing for the survey mode effect on contingent valuation data quality: a case study of web based versus in-person interviews. Ecol Econ 15 Mai.

[14] Kramer LB, Whiteman SE, Petri JM, Spitzer EG, Weathers FW (2023). Self-rated versus clinician-rated assessment of posttraumatic stress disorder: an evaluation of discrepancies between the PTSD checklist for DSM-5 and the clinician-administered PTSD scale for DSM-5. Assessment.

[15] Lenferink LIM, Franzen M, ten Klooster PM, Knaevelsrud C, Boelen PA, Heeke C (2023). The traumatic grief inventory-clinician administered: a psychometric evaluation of a new interview for ICD-11 and DSM-5-TR prolonged grief disorder severity and probable caseness. J Affect Disord Juni.

[16] Boelen PA, Smid GE (2017). The traumatic grief Inventory Self-Report Version (TGI-SR): introduction and preliminary psychometric evaluation. J Loss Trauma 3 April.

[17] Lenferink LIM, Eisma MC, Smid GE, de Keijser J, Boelen PA (2022). Valid measurement of DSM-5 persistent complex bereavement disorder and DSM-5-TR and ICD-11 prolonged grief disorder: the traumatic grief inventory-self Report Plus (TGI-SR+). Compr Psychiatry 1 Januar.

[18] Prigerson HG, Boelen PA, Xu J, Smith KV, Maciejewski PK (2021). Validation of the new DSM-5‐TR criteria for prolonged grief disorder and the PG‐13‐Revised (PG‐13‐R) scale. World Psychiatry Januar.

[19] Rosner R, Comtesse H, Vogel A, Doering BK (2021). Prevalence of prolonged grief disorder. J Affect Disord Mai.

[20] Killikelly C, Lorenz L, Bauer S, Mahat-Shamir M, Ben-Ezra M, Maercker A (2019). Prolonged grief disorder: its co-occurrence with adjustment disorder and post-traumatic stress disorder in a bereaved Israeli general-population sample. J Affect Disord April.

[21] Shevlin M, Redican E, Hyland P, Murphy J, Karatzias T, Mc Bride. O, u. a. symptoms and levels of ICD-11 prolonged grief disorder in a representative community sample of UK adults. Soc PSYCHIATRY Psychiatr Epidemiol; April 2023. 10.1007/s00127-023-02469-1 PMC10098228 37039844

[22] Treml J, Brähler E, Kersting A, Prevalence. Factor structure and correlates of DSM-5-TR criteria for prolonged grief disorder. Front Psychiatry Mai 2022;13. 10.3389/fpsyt.2022.880380 PMC9159802 35664467

[23] Boelen PA, Lenferink LIM, Nickerson A, Smid GE (2018). Evaluation of the factor structure, prevalence, and validity of disturbed grief in DSM-5 and ICD-11. J Affect Disord November.

[24] Killikelly C, Merzhvynska M, Zhou N, Stelzer EM, Hyland P, Rocha J (2021). u. a. examination of the new ICD-11 prolonged grief disorder guidelines across five international samples. Clin Psychol Eur Januar.

[25] Killikelly C, Kagialis A, Henneman S, Coronado H, Demanarig D, Farahani H (2023). u. a. measurement and assessment of grief in a large international sample. J Affect Disord April.

[26] Boelen PA, Lenferink LIM (2022). Prolonged grief disorder in DSM-5-TR: early predictors and longitudinal measurement invariance. Aust N Z J Psychiatry Juni.

[27] Haneveld J, Rosner R, Vogel A, Kersting A, Rief W, Steil R (2022). u. a. same name, same content? Evaluation of DSM-5-TR and ICD-11 prolonged grief criteria. J Consult Clin Psychol April.

[28] Bryant RA, Bawaneh A, Giardinelli L, Awwad M, Al-Hayek H, Akhtar A (2021). A prevalence assessment of prolonged grief disorder in Syrian refugees. World Psychiatry Juni.

[29] Zhou N, Wen J, Stelzer EM, Killikelly C, Yu W, Xu X. Prevalence and associated factors of prolonged grief disorder in Chinese parents bereaved by losing their only child. Psychiatry Res Februar 2020;284. 10.1016/j.psychres.2020.112766 31951871

[30] Baumann I, Künzel J, Goldbeck L, Tutus D, Niemitz M (2022). Prolonged grief, posttraumatic stress, and depression among bereaved parents: prevalence and response to an intervention program. Omega - J Death Dying Januar.

[31] Tang S, Xiang Z (2021). Who suffered most after deaths due to COVID-19? Prevalence and correlates of prolonged grief disorder in COVID-19 related bereaved adults. Glob Health Februar.

[32] Gang J, Falzarano F, She WJ, Winoker H, Prigerson HG (2022). Are deaths from COVID-19 associated with higher rates of prolonged grief disorder (PGD) than deaths from other causes?. Death Stud Januar.

[33] Buur C, Zachariae R, Komischke-Konnerup K, Marello M, Schierff L, O’Connor M. Risk factors for prolonged grief symptoms a systematic review and meta-analysis. Clin Psychol Rev. 2023;102375. 10.1016/j.cpr.2023.102375 38181586

[34] Jordan AH, Litz BT (2014). Prolonged grief disorder: diagnostic, assessment, and treatment considerations. Prof Psychol Res Pract.

[35] Wittouck C, Van Autreve S, De Jaegere E, Portzky G, van Heeringen K (2011). The prevention and treatment of complicated grief: a meta-analysis. Clin Psychol Rev Januar.

[36] Caarls PJ, van Schijndel MA, Kromkamp M, Wierdsma AI, Osse RJ, van Hoeven G (2019). Der, u. a. need analysis for a new high acuity medical psychiatry unit: which patients are considered for admission?. BMC Health Serv Res Februar.

[37] Ziegenbein M, Anreis C, Brüggen B, Ohlmeier M, Kropp S. Januar. Possible criteria for inpatient psychiatric admissions: which patients are transferred from emergency services to inpatient psychiatric treatment? 2021. 10.1186/1472-6963-6-150 PMC1664560 17121672

[38] Carlson EB, Dalenberg C, Armstrong J, Daniels JW, Loewenstein R, Roth D (2001). Multivariate prediction of posttraumatic symptoms in Psychiatric inpatients. J Trauma Stress 1 Juli.

[39] Saba DK, Levit KR, Elixhauser A. Hospital stays related to mental health, 2006. Healthc Cost Util Proj HCUP Stat Briefs Internet; 2008. 21595137

[40] McFarlane AC, Bookless C, Air T (2001). Posttraumatic Stress Disorder in a General Psychiatric Inpatient Population. J Trauma Stress 1 Oktober.

[41] Pratt SI, Rosenberg S, Mueser KT, Brancato J, Salyers M, Jankowski MK (2005). u. a. evaluation of a PTSD psychoeducational program for psychiatric inpatients. J Ment Health 1 April.

[42] Szuhany KL, Malgaroli M, Miron CD, Simon NM (2021). Prolonged grief disorder: Course, diagnosis, Assessment, and treatment. FOCUS 1 Juni.

[43] Margraf J, Cwik JC, Mini. -DIPS Open Access: Diagnostic Short-Interview for Mental Disorders [Internet]. Bochum: Forschungs- und Behandlungszentrum für psychische Gesundheit, Ruhr-Universität; 2017. Available at: 10.13154/rub.102.91.

[44] Margraf J, Cwik JC, Suppiger A, Schneider S (2017). DIPS Open Access: diagnostic interview for Mental disorders.

[45] Margraf J, Cwik JC, Pflug V, Schneider S. Structured clinical interviews for mental disorders across the lifespan: psychometric quality and further developments of the DIPS Open Access interviews. Z Für Klin Psychol Psychother. 2017;(46):176–86.

[46] Derogatis LR. Brief Symptom Inventory (BSI), administration, scoring, and procedures manual. 3rd ed. National Computer Services; 1993.

[47] R Core Team. R: A language and environment for statistical computing. [Internet]. Vienna, Austria: R Foundation for Statistical Computing. 2022. https://www.R-project.org/.

[48] Wei T, Simko V. R package „corrplot: Visualization of a Correlation Matrix [Internet]. 2021. https://github.com/taiyun/corrplot.

[49] Revelle W. psych: Procedures for Psychological, Psychometric, and Personality Research [Internet]. 2023. https://CRAN.R-project.org/package=psych.

[50] Bernaards CA, Jennrich RI (2005). Gradient Projection algorithms and Software for arbitrary rotation criteria in factor analysis. Educ Psychol Meas.

[51] Kaiser HF (1960). The application of electronic computers to factor analysis. Educ Psychol Meas.

[52] Lê S, Josse J, Husson F, FactoMineR (2008). A Package for Multivariate Analysis. J Stat Softw.

[53] Lüdecke D, sjPlot. Data Visualization for Statistics in Social Science [Internet]. 2023. https://CRAN.R-project.org/package=sjPlot.

[54] Wickham H. ggplot2: Elegant Graphics for Data Analysis [Internet]. New York: Springer Verlag; 2016. https://ggplot2.tidyverse.org.

[55] Hayes AF, Coutts JJ (2020). Use Omega rather than Cronbach’s alpha for estimating reliability. Commun Methods Meas 2 Januar.

[56] Gamer M, Lemon J, Puspendra Singh IF. irr: Various Coefficients of Interrater Reliability and Agreement [Internet]. 2019. https://CRAN.R-project.org/package=irr.

[57] Robin X, Turck N, Hainard A, Tiberti N, Lisacek F, Sanchez JC (2011). u. a. pROC: an open-source package for R and S + to analyze and compare ROC curves. BMC Bioinformatics.

[58] Liaw A, Wiener M (2002). Classification and regression by randomForest. R News.

[59] Zwick WR, Velicer WF (1986). Comparison of five rules for determining the number of components to retain. Psychol Bull.

[60] Cacciatore J, Frances A (2022). Prolonged grief disorder – authors’ reply. Lancet Psychiatry 1 September.

[61] Comtesse H, Vogel A, Kersting A, Rief W, Steil R, Rosner R. When does grief become pathological? Evaluation of the ICD-11 diagnostic proposal for prolonged grief in a treatment-seeking sample. Eur J Psychotraumatology Dezember 2020;11(1). 10.1080/20008198.2019.1694348 PMC6968549 32002134

[62] Eisma MC (2023). Prolonged grief disorder in ICD-11 and DSM-5-TR: challenges and controversies. Aust N Z J Psychiatry 1 Juli.

[63] Pauli R, Lang J. Survey Design Moderates Negativity Bias but not Positivity Bias in Self-Reported Job Stress. Eur J Psychol Assess [Internet]. 12. Januar 2024 [cited 17. April 2024]; 10.1027/1015-5759/a000806.

[64] Lundorff M, Holmgren H, Zachariae R, Farver-Vestergaard I, O’Connor M (2017). Prevalence of prolonged grief disorder in adult bereavement: a systematic review and meta-analysis. J Affect Disord April.

[65] Read J, Hammersley P, Rudegeair T (2007). Why, when and how to ask about childhood abuse. Adv Psychiatr Treat Januar.

[66] Sweeney A, Filson B, Kennedy A, Collinson L, Gillard S (2018). A paradigm shift: relationships in trauma-informed mental health services. BJPsych Adv September.

[67] Chapple A, Ziebland S, Hawton K (2015). Taboo and the different death? Perceptions of those bereaved by suicide or other traumatic death. Sociol Health Illn.

[68] Young J, Bailey G, Rycroft P (2004). Family grief and mental health: a systemic, contextual and compassionate analysis. Aust N Z J Fam Ther.

[69] Shear MK, Reynolds CFI, Simon NM, Zisook S, Wang Y, Mauro C (2016). u. a. optimizing treatment of complicated grief: a randomized clinical trial. JAMA Psychiatry July.

[70] Rosner R, Lumbeck G, Geissner E (2011). Effectiveness of an inpatient group therapy for comorbid complicated grief disorder. Psychother Res 1 März.

[71] German Federal Statistical Office (Destatis). Erweitertes Datenangebot auf Basis einer neuen Statistik für Psychiatrie und Psychosomatik. 2023 [cited 6. October 2023]. Available: https://www.destatis.de/DE/Themen/Gesellschaft-Umwelt/Gesundheit/Krankenhaeuser/krankenhaeuser.html.

[72] Polit DF (2014). Getting serious about test–retest reliability: a critique of retest research and some recommendations. Qual Life Res.

[73] McCrae RR, Kurtz JE, Yamagata S, Terracciano A (2011). Internal consistency, retest reliability, and their implications for personality scale validity. Personal Soc Psychol Rev.

