# Supplementary material for: Prolonged grief disorder in an inpatient psychiatric sample: psychometric properties of a new clinical interview and preliminary prevalence
Source: BMC Psychiatry. 2024 May 1;24:333. doi: 10.1186/s12888-024-05784-2 (PMC11064282; doi:10.1186/s12888-024-05784-2)
Supplement: Supplementary file 1 — Supplementary Material 1 [file 12888_2024_5784_MOESM1_ESM.docx]

**Additional File 1. International Interview for Prolonged Grief Disorder according to ICD-11 (I-PGD-11; Internationales Interview zur Diagnostik der Anhaltenden Trauerstörung nach ICD-11)**

| **Interview-Frage** | **Beurteilung** | **ICD-11-Kriterium** |
| --- | --- | --- |
| [PGD_1] Haben Sie eine:n Partner:in, ein Elternteil, ein Kind oder eine andere Ihnen nahestehende Person verloren? | - Ja - Nein | *Trauerfall (Verlust eines Partners, Elternteils, Kindes oder einer anderen nahestehenden Person)* |
| Wenn „Ja“, welche Person? _________________________________  *(Bitte eintragen)* | | |
| Wenn „Ja“, woran ist die Person gestorben? _______________________________  __________________________________________________________________ | | |
| *Wenn die Frage mit „Nein“ beantwortet wird, kann die ATS nicht erfüllt sein, das Interview wird beendet.* | | |
| [PGD_2] Empfinden Sie eine anhaltende und durchdringende Sehnsucht nach der verstorbenen Person?  oder | - Überhaupt nicht - Kaum - Ein wenig - Ziemlich - sehr | *Anhaltende und durchdringende Trauerreaktion charakterisiert durch Sehnsucht nach dem Verstorbenen oder Anhaltende Beschäftigung mit dem Verstorbenen* |
| [PGD_3] Beschäftigen Sie sich anhaltend und durchdringend mit der verstorbenen Person? | - Überhaupt nicht - Kaum - Ein wenig - Ziemlich - sehr |  |
| *Es muss nur eine von zwei Fragen mit „ziemlich“ oder „sehr“ beantwortet werden, damit das B-Kriterium erfüllt ist. Ist das B-Kriterium nicht erfüllt, endet das Interview, die Diagnose der ATS wird nicht vergeben.* | | |
| [PGD_4] Wird diese anhaltende und durchdringende Sehnsucht oder Beschäftigung begleitet von intensivem, emotionalem Schmerz, z.B. | - Überhaupt nicht - Kaum - Ein wenig - Ziemlich - Sehr | *Begleitet von intensivem emotionalem Schmerz (z.B.: Traurigkeit, Schuldgefühle, Ärger, Verleugnung, Schuldzuweisung, Schwierigkeiten den Tod zu akzeptieren, das Gefühl, einen Teil des eigenen Selbst verloren zu haben, Unfähigkeit positive Stimmung zu erleben, emotionale Taubheit, Schwierigkeiten, sich auf soziale oder andere Aktivitäten einzulassen* |
| [PGD_5] Empfinden Sie Traurigkeit?  *(Wenn nötig, bitte spezifizieren, dass es um Traurigkeit während Sehnsucht/ Beschäftigung in Bezug auf die Trauer geht)* | - Überhaupt nicht - Kaum - Ein wenig - Ziemlich - Sehr |  |
| [PGD_6] Haben Sie Schuldgefühle?  *(Wenn nötig, bitte spezifizieren, dass es um Schuldgefühle während Sehnsucht/ Beschäftigung in Bezug auf die Trauer geht)* | - Überhaupt nicht - Kaum - Ein wenig - Ziemlich - Sehr |  |
| [PGD_7] Empfinden Sie Ärger?  *(Wenn nötig, bitte spezifizieren, dass es um Ärger während Sehnsucht/ Beschäftigung in Bezug auf die Trauer geht)* | - Überhaupt nicht - Kaum - Ein wenig - Ziemlich - Sehr |  |
| [PGD_8] Verleugnen/verdrängen Sie den Tod?  *(Wenn nötig, bitte spezifizieren, dass es um Verdrängung während Sehnsucht/ Beschäftigung in Bezug auf die Trauer geht bzw. seit/ aufgrund des Todes)* | - Überhaupt nicht - Kaum - Ein wenig - Ziemlich - Sehr |  |
| [PGD_9] Geben Sie der verstorbenen Person oder anderen die Schuld an dem Tod?  *(Wenn nötig, bitte spezifizieren, dass es um Schuldzuweisung während Sehnsucht/ Beschäftigung in Bezug auf die Trauer/ den Verlust geht bzw. seit/ aufgrund des Todes)* | - Überhaupt nicht - Kaum - Ein wenig - Ziemlich - Sehr |  |
| [PGD_10] Haben Sie Schwierigkeiten, den Tod zu akzeptieren?  *(Wenn nötig, bitte spezifizieren, dass es um Schwierigkeiten der Akzeptanz während Sehnsucht/ Beschäftigung in Bezug auf den Verlust geht bzw. seit/ aufgrund des Todes)* | - Überhaupt nicht - Kaum - Ein wenig - Ziemlich - Sehr |  |
| [PGD_11] Haben Sie das Gefühl, einen Teil Ihrer Selbst verloren zu haben?  *(Wenn nötig, bitte spezifizieren, dass es um das Gefühl einen Teil seiner selbst verloren zu haben während Sehnsucht/ Beschäftigung in Bezug auf die Trauer geht bzw. seit/ aufgrund des Todes)* | - Überhaupt nicht - Kaum - Ein wenig - Ziemlich - Sehr |  |
| [PGD_12] Fühlen Sie sich unfähig, eine positive Stimmung zu erleben?  *(Wenn nötig, bitte spezifizieren, dass es um Unfähigkeit eine positive Stimmung zu erleben während Sehnsucht/ Beschäftigung in Bezug auf die Trauer geht bzw. seit/ aufgrund des Todes)* | - Überhaupt nicht - Kaum - Ein wenig - Ziemlich - Sehr |  |
| [PGD_13] Fühlen Sie sich emotional taub?  *(Wenn nötig, bitte spezifizieren, dass es um emotionale Taubheit während Sehnsucht/ Beschäftigung in Bezug auf die Trauer geht bzw. seit/ aufgrund des Todes)* | - Überhaupt nicht - Kaum - Ein wenig - Ziemlich - Sehr |  |
| [PGD_14] Haben Sie Schwierigkeiten, sich auf soziale oder andere Aktivitäten einzulassen?  *(Wenn nötig, bitte spezifizieren, dass es um Schwierigkeiten, sich auf soziale Aktivitäten einzulassen während Sehnsucht/ Beschäftigung in Bezug auf die Trauer geht bzw. seit/ aufgrund des Todes)* | - Überhaupt nicht - Kaum - Ein wenig - Ziemlich - Sehr |  |
| *Es muss mindestens eins von zehn Symptomen mit „ziemlich“ oder „sehr“ beantwortet werden, um die Diagnose zu vergeben.* | | |
| [PGD_15] Wann ist die Person gestorben?  *(Bitte eintragen)*  [PGD_16] Seit wann haben Sie diese Symptome?  *(Bitte eintragen)*  [PGD_17] Wie lange trauern Menschen in Ihrer Kultur bzw. Ihrer Religion normalerweise?  *(Bitte eintragen)* | - 6 Monate oder länger - Übersteigt die Norm | *Die Trauerreaktion persistiert für eine atypisch langanhaltende Periode (mindestens 6 Monate) und übersteigt die erwartbaren sozialen, kulturellen oder religiösen Normen der Kultur und des Kontextes des Individuums deutlich. Langanhaltende Trauerreaktionen, die in Anbetracht des kulturellen und religiösen Kontextes der Person einer normativen Trauerperiode entsprechen, werden als normale Trauerreaktionen betrachtet und erhalten keine Diagnose* |
| *Die Symptome müssen seit mindestens 6 Monaten vorliegen, um die Kriterien einer ATS zu erfüllen. Wenn die Zeitdauer noch in einem normalen Bereich für die Kultur oder Religion liegt, wird die Diagnose der ATS nicht vergeben.* | | |
| [PGD_18] Erleben Sie durch die eben besprochene Trauersymptomatik bedeutsame Einschränkungen im persönlichen, familiären, sozialen oder beruflichen Bereich? | - Ja - Nein | *Die Einschränkungen verursachen signifikante Beeinträchtigungen in persönlichen, familiären, sozialen, ausbildungsbezogenen, beruflichen oder anderen wichtigen Funktionsbereichen.* |
| *Die Symptome müssen zu bedeutsamen Einschränkungen führen, andernfalls ist die ATS-Diagnose nicht erfüllt.* | | |
| Wenn ja, bitte beschreiben: *___________________________________________*  *_________________________________________________________________* | | |

**Auswertung (Beispiel strikter Algorithmus)**

| **Gesamtsummenwert (Summe der Items PGD_2 bis PGD_14):**  (Überhaupt nicht = 1, Kaum = 2, Ein wenig = 3, Ziemlich = 4, Sehr = 5) | **___________________________**  (Vorläufiger Cutoff: 32.5, Range 13-65) | |
| --- | --- | --- |
| **Kriterium** | **Erfüllt** | |
| 1. Verlust einer nahestehenden Person | - Ja | - Nein |
| 1. Anhaltende & durchdringende Sehnsucht oder Beschäftigung   (Frage 2 ODER 3 mit „ziemlich“ oder „sehr“ kodiert) | - Ja | - Nein |
| 1. Begleitet von intensivem, emotionalem Schmerz   (mind. 1 von Items 4-13 mit „ziemlich“ oder „sehr“ kodiert) | - Ja | - Nein |
| 1. Dauer   (mind. 6 Monate; nicht erfüllt, wenn die Dauer in der Kultur oder Religion üblich ist) | - Ja | - Nein |
| 1. Bedeutsame Einschränkungen durch den Verlust | - Ja | - Nein |
