# Supplementary material for: Prolonged grief disorder in an inpatient psychiatric sample: psychometric properties of a new clinical interview and preliminary prevalence
Source: BMC Psychiatry. 2024 May 1;24:333. doi: 10.1186/s12888-024-05784-2 (PMC11064282; doi:10.1186/s12888-024-05784-2)
Supplement: Supplementary file 2 — Supplementary Material 2 [file 12888_2024_5784_MOESM2_ESM.docx]

**Additional File 2. English translation of the International Interview for Prolonged Grief Disorder according to ICD-11 (I-PGD-11)**

| **Interview-Question** | **Evaluation** | **ICD-11-Criterion** |
| --- | --- | --- |
| [PGD 1] Have you lost your partner, parent, child or another person who was very close to you? | - Yes - No | *Death of a partner, parent, child, or other person close to the bereaved* |
| If „Yes“, which person? _________________________________  *(Please fill out)* | | |
| If “Yes”, under which circumstances did they die? __________________________  __________________________________________________________________ | | |
| *Must be answered with “yes”, otherwise the diagnosis of PGD cannot be assigned, interview ends.* | | |
| [PGD 2] Do you feel a persistent and pervasive longing for [the deceased]?  or | - Not at all - Slightly - Somewhat - Quite a bit - Overwhelmingly | *Persistent and pervasive grief response characterized by longing for the deceased or persistent preoccupation with the deceased* |
| [PDG 3] Are you persistently and pervasively preoccupied with [the deceased]? | - Not at all - Slightly - Somewhat - Quite a bit - Overwhelmingly |  |
| *At least one of the two questions must be answered with “quite a bit” or “overwhelmingly” otherwise the interview ends* | | |
| [PGD 4] Is this persistent and pervasive longing/ preoccupation accompanied by intense emotional pain, e.g.: | - Not at all - Slightly - Somewhat - Quite a bit - Overwhelmingly | *Accompanied by intense emotional pain (e.g. sadness, guilt, anger, denial, blame, difficulty accepting the death, feeling one has lost a part of one’s self, an inability to experience positive mood, emotional numbness, difficulty in engaging with social or other activities)* |
| [PGD 5] Do you feel sadness?  *(If necessary, please specifiy that you mean sadness during the longing/ preoccupation regarding the grief)* | - Not at all - Slightly - Somewhat - Quite a bit - Overwhelmingly |  |
| [PGD 6] Do you feel guilt?  *(If necessary, please specifiy that you mean guilt during the longing/ preoccupation regarding the grief)* | - Not at all - Slightly - Somewhat - Quite a bit - Overwhelmingly |  |

| [PGD 7] Do you feel anger?  *(If necessary, please specify that you mean anger during the longing/ preoccupation regarding the grief)* | - Not at all - Slightly - Somewhat - Quite a bit - Overwhelmingly |  |
| --- | --- | --- |
| [PGD 8] Are you in denial about [their] death?  *(If necessary, please specify that you mean denial during the longing/ preoccupation regarding the grief or since/ because they died)* | - Not at all - Slightly - Somewhat - Quite a bit - Overwhelmingly |  |
| [PGD 9] Do you feel like [the person you’ve lost] or other people are to blame for [their] death?  *(If necessary, please specify that you mean blame during the longing/ preoccupation regarding the grief or since because they died)* | - Not at all - Slightly - Somewhat - Quite a bit - Overwhelmingly |  |
| [PGD 10] Do you have difficulties accepting the death?  *(If necessary, please specify that you mean acceptance-difficulties during the longing/ preoccupation regarding the loss or since because they died)* | - Not at all - Slightly - Somewhat - Quite a bit - Overwhelmingly |  |
| [PGD 11] Do you feel like you have lost a part of yourself?  *(If necessary, please specify that you mean the feeling of having lost a part of oneself during the longing/ preoccupation regarding the grief or since/ because they died)* | - Not at all - Slightly - Somewhat - Quite a bit - Overwhelmingly |  |
| [PGD 12] Do you feel unable to experience a positive mood?  *(If necessary, please specify that you mean inability to experience positive mood during the longing/ preoccupation regarding the grief or since/ because they died)* | - Not at all - Slightly - Somewhat - Quite a bit - Overwhelmingly |  |
| [PGD 13] Do you feel emotionally numb?  *(If necessary, please specify that you mean emotional numbness during the longing/ preoccupation regarding the grief or since/ because they died)* | - Not at all - Slightly - Somewhat - Quite a bit - Overwhelmingly |  |
| [PGD 14] Do you experience difficulties engaging with social or other activities?  *(If necessary, please specify that you mean social withdrawal during the longing/ preoccupation regarding the grief or since because they died)* | - Not at all - Slightly - Somewhat - Quite a bit - Overwhelmingly |  |
| *At least one of ten must be answered with “quite a bit” or “overwhelmingly” otherwise the interview ends* | | |
| [PGD 15] When did [the person] die?  *(Please fill out)*  [PGD 16] How long have you had these symptoms?  *(Please fill out)*  [PGD 17] How long do people in your culture or religion normally grieve?  *(Please fill out)* | - At least 6 months - Longer/ more intense than expected by culture or religion | *The grief response has persisted for an atypically long period of time following the loss (more than 6 months at a minimum) and clearly exceeds expected social, cultural or religious norms for the individual’s culture and context. Grief reactions that have persisted for longer periods that are within a normative period of grieving given the person’s cultural and religious context are viewed as normal bereavement responses and are not assigned a diagnosis.* |
| *The symptoms must have a duration of at least 6 months. If the given time period is normative for the religion or culture of the interviewee the diagnosis of PGD will not be assigned.* | | |
| [PGD 18] Did these symptoms that were just discussed cause significant impairment in personal, family, social, educational, occupational or other areas of functioning? | - Yes - No | *The disturbance causes significant impairment in personal, family, social, educational, occupational or other important areas of functioning.* |
| *Must be answered with yes for the PGD diagnosis* | | |
| If “Yes”, please describe: _____________________________________________ | | |

**Evaluation (Example strict algorithm)**

| **Score (Sum of Items PGD_2 to PGD_14):**  (Not at all = 1, Slightly = 2, Somewhat = 3, Quite a bit = 4, Overwhelmingly = 5) | _______________________  (Preliminary Cutoff: 32.5, range 13 – 65) | |
| --- | --- | --- |
| **Criterion** | **Fulfilled** | |
| 1. Loss of a person close to the bereaved | - Yes | - No |
| 1. Persistent and pervasive longing or preoccupation with the deceased   (Question 2 or 3 answered with “quite a bit” or “overwhelmingly”) | - Yes | - No |
| 1. Accompanied by intense emotional pain   (At least one of the items 4-13 answered with “quite a bit” or “overwhelmingly”) | - Yes | - No |
| 1. Duration   (at least 6 months; not fulfilled if duration still normative in religion or culture) | - Yes | - No |
| 1. Functional impairment | - Yes | - No |
